# Association of Demographic Variables with the Awareness of Type 2 Diabetes Mellitus Patients (T2DM) among the Northwest Population in Saudi Arabia

**DOI:** 10.1155/2020/9408316

**Published:** 2020-07-12

**Authors:** Wael Ahmed Al Arawi, Udai Salamh Al Shaman, Waleed Ahmad Mohsin Albalawi, Palanisamy Amirthalingam Siddhachettiar, Sherif M. H. El-kannishy, Alaa Bagalagel, Reem Diri, Ahmed Aljabri, Ahmed Mohsen Hamdan

**Affiliations:** ^1^Department of Quality Assurance, Tabuk Pharmaceutical Manufacturing Company, Almadina Road, Tabuk, Saudi Arabia; ^2^Department of Pharmacy Practice, Faculty of Pharmacy, University of Tabuk, Tabuk, Saudi Arabia; ^3^Department of Pharmacology and Toxicology, Faculty of Pharmacy, University of Tabuk, Saudi Arabia; ^4^Department of Toxicology, Emergency Hospital, Faculty of Medicine, Mansoura University, Mansoura, Egypt; ^5^Department of Pharmacy Practice, Faculty of Pharmacy, Kind Abdulaziz University, Jeddah, Saudi Arabia

## Abstract

The chronic hyperglycemia in diabetes is associated with long-term damage, dysfunction, and failure of different organs. Lack of patient education and knowledge about these complications can worsen the quality of a patient's life. Hence, more efforts are needed to improve patient's education especially in rural areas. *Aim*. Our objective is to explore the association between demographic variables and the knowledge of self-care practices in type 2 diabetes mellitus. *Methods*. We used observational cross-sectional descriptive study using a validated self-administered questionnaire in both Arabic and English languages as well. A descriptive correlation design analyzed the questionnaire completed by a convenience sample meeting the inclusion criteria. *Results*. A total of 100 patients met the inclusion criteria for the analysis out of 3251 patients who completed the questionnaire. The study population has low moderate knowledge in diabetes, moderate knowledge in self-care practices, and good knowledge about complications of nephropathy and cardiovascular disease. No significant association between demographic variables. However, better knowledge observed in male (*p* = 0.028) and self-care practices with female (*p* = 0.020). Further, educational status is significantly influencing the knowledge of diabetic patients. *Conclusion*. The study emphasizing irrespective of demographic variable and the importance of patient education to achieve well glycemic control.

## 1. Introduction

Type 2 diabetes mellitus (T2DM) is a chronic metabolic disorder affecting about 425 million people worldwide in 2017, and, according to the International Diabetes Federation (IDF) report, is expected to affect up to 629 million people by 2045 [1]. Saudi Arabia has the thirteenth highest prevalence of diabetes for adults 20–79 years old [2]. Saudi Arabia is the fourth highest prevalence rate of T2DM compared to other Arabic-speaking countries [3]. T2DM can be controlled by adhering to self-care practices, including blood glucose monitoring, diet, exercise, foot care, eye care, and medication [4], as well as with regular follow-up with health care providers [5]. Secondary diseases due to ineffective self-management of T2DM, such as diabetic foot ulcers and neuropathy, can potentially further diminish a patient's quality of life [6]. The prevalence of diabetes mellitus (DM) is increasing dramatically, placing a considerable financial burden on the healthcare budget of each country. Patient self-management is crucial for the control of blood glucose, which largely determines the chances of developing diabetes-related complications. Self-management interventions vary widely, and a method is required for assessing the impact of self-management. The Kingdom of Saudi Arabia had achieved a notable economic growth and improvement in life quality during the past three decades. The population of the country had experienced a remarkable change in lifestyles and hence increasing rate of noncommunicable disease. Recent changes in the physical activities and dietary patterns have promoted the development of diabetes in KSA [7]. If different preventive and control activities are not adopted, by the year 2025, more than 9 million Saudi people (13% of the population above 20 years old) will have diabetes [8]. Diabetes education, with consequent improvement in knowledge, attitudes, and skills, leads to better control of the disease and is widely accepted to be an integral part of comprehensive diabetes care [9]. To the best of authors' knowledge, no previous studies have been done in rural Tabuk, Saudi Arabia, where the situation appears to be different than any other place in Saudi Arabia. There are two previous studies for the knowledge, attitude, and practices for diabetes mellitus in Saudi Arabia in Riyadh and Hail regions [10, 11]. The objectives of the study were to assess knowledge, attitude, practice, and compliance of diabetic patients in public, to identify the predictors of good level of knowledge and patient's compliance and to measure the impact of self-management in diabetes using a designed knowledge, attitude, and practice (KAP) questionnaire on selected patients (late adulthood (51 to 64 years old) and young old age (65 to 74 years old)). We assumed that the newly established University of Tabuk (since 2006) and the nutritional center at King Fahd Specialized Hospital have a great role in improving the public knowledge for diabetic patients in this rural area.

## 2. Subjects

Patients with T2DM were included in the study and given a predesigned questionnaire completed during face-to-face interviews. We randomly selected participants over the age of 18 years (both males and females) at different locations in large primary care centers and private pharmacies in the Tabuk region. We excluded young people (younger than 18 years old) and lately diagnosed diabetic patients (before less than 5 years). Moreover, we excluded patients who had bad handwritings, who intended to hide information, who did not understand the questions even after explanation, who gave irrelevant information, who are mentally handicapped, who are with medical background (all health-related education), who had attended diabetes education programs in the last 6 months, who were on a hurry, and who did not complete the questionnaire. Subjects were consented to participate after they were given full details of the study and its intended aims. Moreover, we clarified their queries before obtaining the consent. Patients fulfilled the inclusion criteria (aged between 51 and 74 years; were classified into (late adulthood: 51 to 64 years old, and young old age: 65 to 74 years old)) and were included for the final analysis. The rationale for choosing the areas is to observe the transition of the disease as a consequence of changing lifestyles and the effect of the presence of both the university and diabetic center related to the ministry of health in Tabuk as well. Besides, these areas were included as 2.77% of the total population of Saudi live in such areas according to the 2019 national statistics [12].

## 3. Material and Methods

### 3.1. Study Design and Sampling

An observational cross-sectional descriptive study extended for one year from March 2018 to September 2019. We used a previously validated questionnaire for the knowledge, attitude, and practice for diabetes mellitus in both Arabic and English languages as well. The Arabic translation was done by a professional Arabic speaker and expert in both languages with good explaining ability for the meaning to the publics. The Arabic version then went through further revision and then back translated into English by another bilingual translator. This Arabic version of the final form was examined and approved by two diabetologists to assess content and construct validity. The questionnaire did not contain any questions which can reveal the identity of patients or their treating doctors. The first segment of the questionnaire included the socio-demographics of the participants. Data included gender, age, level of education, average income, and occupation. Participants were also asked whether their occupation and education were related to the medical field. Knowledge regarding T2DM was measured using eight main questions related to the risk factors, diagnosis, prevention, and complications of diabetes mellitus. The possible categorical answers were “Yes,” “Do not know,” and “No.” The diabetes attitude was assessed using seven questions related to compliance with the treatment of DM. The possible categorical answers were “Yes,” “Do not know,” and “No.” Patients' interviews were conducted at the outpatient departments (OPDs) of King Fahd Specialized Hospital in Tabuk and attended twelve different large private community pharmacies in Tabuk region. The sample size was calculated using alpha error 5% and study power 80%, a test value (%) of 11%, and expected sample value (%) of 14% giving a sample size of 100 with adding 10% for nonresponse rate a final sample of 100 was calculated. Participants were well informed about the study and also clarified their queries before obtaining the consent. Out of 325 participants with type 2 diabetes mellitus showed willingness to participate; however, only 100 participants fulfilled the inclusion criteria (aged between 51 and 74 years; we classified them into (late adulthood: 51 to 64 years old, and young old age: 65 to 74 years old)) and were included in the study.

### 3.2. Data Collection and Analysis

Before administering questionnaires, participants were orally informed about the purpose of the study, emphasizing that participation was voluntary and that their answers would be kept confidential. A structured and pretested data collection form to inquire about such as age, sex, residence, education, occupation, past-medical history, past medication history, and social. A questionnaire used to collect the information from the respondents is consisting four segments including the socio-demographic data, the knowledge of diabetes, self-care practices, and complications [13, 14]. A pilot study was carried out on 20 diabetic patients (not included in the final analysis) to test our tool and make necessary modifications. Data were analyzed using SPSS version 20. Chi-squared test/Fisher's exact were used for comparison between groups. Significant variables on univariate analysis were entered into multivariate logistic regression analysis. Respondent's socio-demographic characteristics were stated using descriptive statistics. Means, standard deviations, and proportions were generated to describe the overall sample characteristics (age, gender, occupational status, disease duration, and education). Student's *t*-test and ANOVA were used to test the equality of means between demographic variables.

### 3.3. Ethics Approval

The study protocol was approved by the Local Research Ethics Committee of the University of Tabuk under the number (UT-64-26-2018), including the use of oral consent for the collection of data. Meanwhile, the Institutional Review Board (IRB) of our institution exempted our study from review. The committee unconditionally approved the project and agreed to verbal consent to be used.

## 4. Results

### 4.1. Patient Demographics

During our study, 3786 males and 3831 females were subjected to the preliminary analysis. The percent of T2DM (4365 patients) from the totally analyzed patients was about 57.3%. 3251 patients, from those showed willingness to participate (74.48% response rate), were included in this study and were given a questionnaire using the purposive sampling method. Only 100 patients fulfilled the inclusion criteria (aged between 51 and 74 years; we classified them into (late adulthood: 51 to 64 years old, and young old age: 65 to 74 years old)) and were included for the final analysis. In addition to KAP, we collected socio-demographic data that include gender, age, occupation, marital status, educational level, income, family history of diabetes, duration of diabetes, and medications. The details of patient demographics mentioned in Table 1. Among 100 participants 52 (52%) were male. A view number of participants 9 (1.72%) were illiterate, whereas 25 (48%) completed primary education and 33 (63.4%) participants were graduate. Of the 100 participants, about 86 (86%) identified their type of diabetes and know that it is a lifestyle disease. As showed in Table 1, 69% (69) of the patients were between 51 and 64 years old and 31% (31) 65-74 years age group, respectively. 46 patients (46%) and 43 patients (43%) were suffering from diabetes for 0-5 years and 6-10 years, respectively. 61% of the study population were unemployed. Overall, moderate knowledge observed among the study population regarding the knowledge, self-care practices, and complications of diabetes. However, most of the study population well aware bout the T2DM is a lifestyle-related disease, self-monitoring of blood glucose, foot care, and complications of kidney disease and ischemic heart disease. On the other hand, poor knowledge on need of monitoring both fasting and postprandial blood sugar level and high fiber in diet; also, poor awareness about the complications includes eye disease and stroke.

### 4.2. Difference between the Demographic Variables

Student's *t*-test (Tables 2 and 3) shows no significant difference between the age group, gender, and occupational status; however, female found to have better knowledge significantly than the male regarding diabetes is a lifestyle disease (*p* = 0.028), awareness of self-monitoring of blood glucose level in diabetes (*p* = 0.020), and uncontrolled diabetes can cause stroke (*p* = 0.034). The analysis of ANOVA test results (Tables 4 and 5) reflects that the no significant difference among the diabetes disease duration and educational status affects knowledge of diabetes about only in diabetes is a lifestyle disease (*p* ≤ 0.001) and awareness about HbA1c apart from blood glucose (*p* = 0.038).

### 4.3. Multivariate Analysis

As shown in Table 6, multiple linear regressions for the association of knowledge score on covariates identified in multivariate analysis showed no significant association among the age group, disease duration, and occupational status. However, the regression analysis showed no significant association between age, occupation status, and disease duration with knowledge of diabetes, self-care practices and knowledge regarding complications of diabetes. Also, there was no significant association between the gender and educational status with knowledge of diabetes. However, after adjusting the respondent's characteristic variables, significant association among the female gender with the awareness of self-monitoring of blood glucose level in diabetes (*p* = 0.036) and knowledge on uncontrolled diabetes can cause stroke (*p* = 0.026). Further, educational status has significant association with the importance of foot care in DM (*p* = 0.037).

## 5. Discussion

Saudi Arabia is ranked the second country in the Middle East and the seventh country worldwide for the rate of diabetes [2]. This high popularity and the correlated comorbidities and burdens lead to the necessity for health caregivers to authorize prevention and management programs for diabetes. Knowledge, attitudes, and practice studies on the targeted population are the cornerstone for these prevention and management plans. Though many studies already established knowledge, attitude, and practice in DM, to the best of our knowledge, this is a first study about the assessment knowledge, self-care practices, and complications of DM among the population in the north-west region of Saudi Arabia. Our principle objective in this study is to investigate the perception of normal patient on knowledge, self-care practice, and complications of diabetes among the northwest population; the region showing high incidence of type 2 diabetes mellitus in Saudi Arabia. We calculated the sample size. Then, we made an observational cross-sectional study. So, the exclusion criteria for the characteristics is the extra normal or the unreal characteristics of normal patients which include patients with bad handwritings, who intended to hide information, who did not understand the questions even after explanation, who gave irrelevant information, who are mentally handicapped, who are with medical background (all health-related education), who had attended diabetes education programs in the last 6 months, and who were on hurry and who did not complete the questionnaire. This investigation will give a real study for the current situation for their normal knowledge and self-caring practice. We found only 100 patients included (3%) of the sample. We choose this age limit due to different reasons. First, Prevalence of diabetes mellitus type 2 is mentioned to be high in Saudi Arabia in this age group [15, 16]. Second, people aged over 50 years old showing high incidence of diabetic complications with less family care and self-supported for their diabetes management. They show functionally independent and rely on their own [17]. Third, according to Nazar et al., most diabetic patients with type 2 diabetes mellitus are over 60 with poor level of diabetes knowledge [18]. So, they should be targeted for diabetic education. Finally, National Health Check (NHC) for diabetes globally is between 40 and 74 years old [19].

The studied population in the present study found to have a low to moderate knowledge about the disease factors, which is consistent with the recent systematic review published from Saudi Arabia and they addressed poor knowledge of health care professionals in this regard [13–21]. The importance of patient education already been established in the studies conducted in the United Kingdom [14, 15] and India [15]. However, this association of knowledge, self-care practices, and complications of DM with different demographic variables among the respondents since they have a better literacy rate (91%), developed social networks since the study was carried out in urban area. These reports substantiate the recently published reports in Hail region [12], Riyadh [10, 11, 21, 22] Saudi Arabia, and further acknowledged by the other foreign studies, those found to have poor knowledge of DM among the rural population reported in India [23], Pakistan [24], Sudan [25], and Malaysia [26]. Meanwhile, the other studies distributed the questionnaires through electronic media and different social media platforms. Such distribution may lead to false high results due to submission of many responses. Yet, they did not take into consideration their recent training for some patients. Here, we performed our study using interviewed patients in health care facilities. Such patients are seeking for medical advice. So, we assessed their knowledge, attitude, and practice towards this disease. Besides, we took in our consideration many factors affecting the public knowledge and practices to give a real image about the situation in these rural areas. Male and female has significantly different good knowledge than each other on the awareness of diabetes is lifestyle-related disease and self-monitoring of blood glucose (*p* = 0.028 and 0.02, respectively). This result is more reliable than the previously published report [27]. A significant influence of educational status with the knowledge of diabetes is inconsistent with the previous report [28]. Female gender seems to have significant better knowledge for the association with the awareness for the complication of type 2 diabetes mellitus. The attendance of diabetic education program is significantly (*p* = 0.02) higher in female [12]. Willing to participate in such events might be the factor for better knowledge for the complication of the disease among females than males in Saudi Arabia. Meanwhile, we noticed that there is no significant difference between genders for their knowledge about the disease. This may be due to female contact to the social media and the educational programs held by the University of Tabuk and the diabetic center. So, the female gender showing advanced knowledge and self-practicing towards type 2 diabetes mellitus. The two references kindly referred by the reviewer show the follow-up intervention within six months after self-management support program within the first year of illness, which we excluded in our study. We investigated the long term self-management practices for diabetic patients. Yet, we recommend different educational strategies directed for males such as to group discussion education, health campaigns, and booklets.

Moderate score on self-care practices observed with no significant influence on the demographic variables including age, gender, disease duration, and occupational status. The influence of socioeconomic factors in self-care practices of diabetes is already established by the previous investigators [29, 30]. Socioeconomic status inequality is recently reported in Saudi Arabia [31], and also chronic health problems and self-health perception were considered to have a significant factor in this regard [32]. Nevertheless, these factors have no effect in the present study, since there are no validated indices available to measure socioeconomic status in Saudi Arabia. Moreover, knowledge of diabetes does not reflect on self-care practices in diabetes [33]. In the complications of diabetes, the study population has good knowledge on the association of uncontrolled diabetes with kidney and heart diseases; meanwhile, there is poor knowledge on eye disease and stroke. The association of poor knowledge on retinopathy [34, 35] and stroke [36] already established. However, these microvascular and macrovascular complications considered as a challenge in the management of diabetes in Saudi Arabia [36]. Many education programs warranted for both the patients and physicians to enhance the patient awareness with regard to knowledge, self-care practices, and complications of diabetes mellitus and to achieve glycemic control in long term care [27, 37]. The diabetic patients with lower education should receive ongoing need-based quality diabetic education by using innovative methods that are tailored to their needs delivered by skilled health care providers. This will be the role of the University of Tabuk and the diabetic centers to increase such awareness and improve the self-caring practices.

## 6. Strength and Limitations

This is a preliminary attempt to investigate the patient perceptions on knowledge, self-care practice, and complications of diabetes among the northwest population in Saudi Arabia. This study was also limited to diabetic patients who were interested to participate. We assessed their knowledge, attitude, and practice on disease, self-care practice, complications, and insulin use. Moreover, we used a well-documented questionnaire for the diabetic knowledge.

## 7. Conclusion

The present study recommends the need of patient education to attain medication adherence; thus, it brings better glycemic control in order to protect this population from complications of diabetes mellitus. The pharmacist may play pivotal role in educating the diabetic population. The study showed moderate level of diabetes awareness and good level of positive attitudes towards self-care practice. There is a need to carry out large-scale awareness programs to spread the message to the general population.

## Figures and Tables

**Table 1:** Demographics of the study population.

Parameter	Number of patients	% of patients
Gender		
Male	52	52
Female	48	48
Age		
Late adulthood (51 to 64)	69	69
Young old age (65 to 74)	31	31
Duration of diabetes		
5 to 10 years	46	46
10 to 15 years	43	43
More than 15 years	11	11
Education		
Illiteracy	9	9
Primary	25	25
High school	58	58
College	8	8
Social history		
Smokers	30	30
Nonsmokers	70	70
Occupation		
Workers	39	39
Nonworkers	61	61

**Table 2:** Association of attitude of respondents towards the knowledge and level of care related to diabetes and its management with age and gender.

Questions	Overall	Age (mean ± SD)				Gender			
		Late adulthood (*n* = 69)	Young old age (*n* = 31)	*t*	*p*	Male (*n* = 52)	Female (*n* = 48)	*t*	*p*
Knowledge of diabetes									
Is diabetes mellitus a lifestyle-related disease?	1.80 ± 0.50	1.814 ± 0.441	1.71 ± 0.643	1.184	0.239	1.92 ± 0.347	1.69 ± 0.612	-2.231	**0.028**
Is it important to do both fasting as well as postprandial blood sugar level for diagnosis and monitoring?	0.7 ± 0.9	0.78 ± 0.921	0.52 ± 0.851	0.174	0.266	0.81 ± 0.908	0.58 ± 0.895	1.243	0.217
Apart from blood sugar level, are you aware about HbA1c?	0.97 ± 0.97	0.96 ± 0.977	1.00 ± 1.00	-0.204	0.840	1.04 ± 0.969	0.90 ± 0.994	0.726	0.470

Self-care practices									
Are you aware of self-monitoring of blood glucose level in diabetes?	1.64 ± 0.74	1.61 ± 0.732	1.71 ± 0.693	-0.649	0.518	1.48 ± 0.804	1.81 ± 0.571	-2.361	**0.020**
Is it necessary to consume high fiber diet in DM care?	1.12 ± 0.93	1.09 ± 0.919	1.19 ± 0.980	-0.525	0.601	1.12 ± 0.922	1.13 ± 0.959	-0.051	0.959
Is foot care necessary in DM?	1.61 ± 0.71	1.62 ± 0.709	1.58 ± 0.765	0.271	0.787	1.50 ± 0.804	1.73 ± 0.610	-1.595	0.114

Knowledge regarding complications of DM									
Unmanaged DM can cause eye problems or even blindness	0.94 ± 0.97	1.03 ± 0.985	0.74 ± 0.965	1.356	0.178	0.94 ± 0.978	0.94 ± 0.998	0.024	0.981
Uncontrolled DM can affect your kidneys	1.64 ± 0.74	1.61 ± 0.771	1.71 ± 0.693	-0.624	0.534	1.58 ± 0.801	1.71 ± 0.683	-0.879	0.381
Uncontrolled DM can cause ischemic heart disease.	1.55 ± 0.79	1.52 ± 0.815	1.61 ± 0.761	-0.528	0.599	1.48 ± 0.852	1.63 ± 0.733	-0.904	0.368
Uncontrolled DM can cause stroke	0.62 ± 0.83	0.59 ± 0.828	0.68 ± 0.871	-0.457	0.648	0.79 ± 0.915	0.44 ± 0.712	2.129	**0.034**

Student's t-test; two-tailed *p* < 0.05 is considered as significant.

**Table 3:** Association of attitude of respondents towards the knowledge and level of care related to diabetes and its management with education.

Questions	Demographic variables			
	Occupational status (mean ± SD)			
	Working (*n* = 69)	Not working (*n* = 31)	*t*	*p*
Knowledge of diabetes				
Is diabetes mellitus a lifestyle-related disease?	1.85 ± 0.366	1.77 ± 0.589	0.516	0.474
Is it important to do both fasting as well as postprandial blood sugar level for diagnosis and monitoring?	0.77 ± 0.959	0.66 ± 0.873	0.372	0.543
Apart from blood sugar level, are you aware about HbA1c?	0.95 ± 0.972	0.98 ± 0.991	0.030	0.863

Self-care practices				
Are you aware of self-monitoring of blood glucose level in diabetes?	1.64 ± 0.743	1.64 ± 0.708	**≤0.001**	0.991
Is it necessary to consume high fiber diet in DM care?	1.18 ± 0.942	1.08 ± 0.936	0.257	0.613
Is foot care necessary in DM?	1.59 ± 0.751	1.62 ± 0.711	0.050	0.824

Knowledge regarding complications of DM				
Unmanaged DM can cause eye problems or even blindness	0.95 ± 0.999	0.93 ± 0.981	0.005	0.944
Uncontrolled DM can affect your kidneys	1.67 ± 0.737	1.62 ± 0.756	0.081	0.777
Uncontrolled DM can cause ischemic heart disease.	1.67 ± 0.737	1.48 ± 0.829	1.378	0.243
Uncontrolled DM can cause stroke	0.56 ± 0.852	0.66 ± 0.834	0.282	0.596

Student's *t*-test; two-tailed *p* < 0.05 is considered as significant.

**Table 4:** Association of attitude of respondents towards the knowledge and level of care related to diabetes and its management with disease duration.

Questions	Disease duration (mean ± SD)				
	0-5years (*n* = 46)	6-10 years (*n* = 42)	>10years (*n* = 12)	*F*	*p*
Knowledge of diabetes					
Is diabetes mellitus a lifestyle-related disease?	1.76 ± 0.565	1.88 ± 0.395	1.67 ± 0.651	1.066	0.348
Is it important to do both fasting as well as postprandial blood sugar level for diagnosis and monitoring?	0.80 ± 0.957	0.57 ± 0.831	0.75 ± 0.965	0.745	0.478
Apart from blood sugar level, are you aware about HbA1c?	1.02 ± 0.977	0.86 ± 1.002	1.17 ± 0.937	0.580	0.562

Self-care practices					
Are you aware of self-monitoring of blood glucose level in diabetes?	1.65 ± 0.737	1.71 ± 0.596	1.33 ± 0.985	1.335	0.268
Is it necessary to consume high fiber diet in DM care?	1.15 ± 0.918	1.14 ± 0.952	0.92 ± 0.996	0.319	0.728
Is foot care necessary in DM?	1.57 ± 0.720	1.74 ± 0.627	1.33 ± 0.985	1.646	0.198

Knowledge regarding complications of DM					
Unmanaged DM can cause eye problems or even blindness	1.11 ± 0.994	0.83 ± 0.961	0.67 ± 0.985	1.400	0.252
Uncontrolled DM can affect your kidneys	1.65 ± 0.737	1.62 ± 0.764	1.67 ± 0.778	0.030	0.971
Uncontrolled DM can cause ischemic heart disease.	1.54 ± 0.808	1.52 ± 0.804	1.67 ± 0.778	0.150	0.860
Uncontrolled DM can cause stroke	0.67 ± 0.871	0.57 ± 0.801	0.58 ± 0.900	0.174	0.840

One way ANOVA test; two-tailed *p* < 0.05 is considered as significant.

**Table 5:** Association of attitude of respondents towards the knowledge and level of care related to diabetes and its management with education.

Questions	Education status (mean ± SD)					
	Illiterate (*n* = 8)	Primary (*n* = 25)	High school (*n* = 59)	College (*n* = 8)	*F*	*p*
Knowledge of diabetes						
Is diabetes mellitus a lifestyle-related disease?	1.13 ± 0.991	1.50 ± 0.756	1.88 ± 0.332	1.90 ± 0.357	7.799	**≤0.001**
Is it important to do both fasting as well as postprandial blood sugar level for diagnosis and monitoring?	0.50 ± 0.535	0.64 ± 0.907	0.73 ± 0.944	0.88 ± 0.991	0.281	0.839
Apart from blood sugar level, are you aware about HbA1c?	0.72 ± 0.980	0.92 ± 0.988	1.38 ± 0.916	1.75 ± 0.463	2.913	**0.038**

Self-care practices						
Are you aware of self-monitoring of blood glucose level in diabetes?	1.25 ± 1.035	1.50 ± 0.756	1.64 ± 0.713	1.80 ± 0.577	1.315	0.274
Is it necessary to consume high fiber diet in DM care?	0.50 ± 0.756	1.00 ± 1.069	1.17 ± 0.913	1.24 ± 0.970	1.427	0.240
Is foot care necessary in DM?	1.00 ± 0.756	1.63 ± 0.740	1.68 ± 0.690	1.88 ± 0.354	2.447	0.69

Knowledge regarding complications of DM						
Unmanaged DM can cause eye problems or even blindness	0.80 ± 0.957	0.86 ± 0.991	1.38 ± 0.916	1.50 ± 0.926	1.709	0.170
Uncontrolled DM can affect your kidneys	1.25 ± 0.886	1.58 ± 0.814	1.88 ± 0.440	1.75 ± 0.707	1.840	0.145
Uncontrolled DM can cause ischemic heart disease.	1.25 ± 0.886	1.51 ± 0.817	1.50 ± 0.926	1.76 ± 0.663	1.023	0.386
Uncontrolled DM can cause stroke	0.60 ± 0.816	0.58 ± 0.814	0.75 ± 1.035	0.88 ± 0.991	0.362	0.781

One way ANOVA test; two-tailed *p* < 0.05 is considered as significant.

**Table 6:** Association of respondents' knowledge with the demographic variables.

Questions	Demographic variables									
	Age		Gender		Educational status		Occupation		Disease duration	
	*R* ^2^	*p*	*R* ^2^	*p*	*R* ^2^	*p*	*R* ^2^	*p*	*R* ^2^	*p*
Knowledge of diabetes										
Is diabetes mellitus a lifestyle-related disease?	0.004	0.239	0.023	0.072	0.019	0.090	-0.005	0.474	-0.006	0.531
Is it important to do both fasting as well as postprandial blood sugar level for diagnosis and monitoring?	0.009	0.174	0.005	0.217	-0.022	0.362	-0.006	0.543	-0.005	0.498
Apart from blood sugar level, are you aware about HbA1c?	-0.010	0.893	-0.003	0.414	-0.002	0.384	-0.010	0.964	-0.010	0.845

Self-care practices										
Are you aware of self-monitoring of blood glucose level in diabetes?	-0.008	0.636	0.035	**0.036**	0.003	0.415	-0.009	0.714	-0.009	0.745
Is it necessary to consume high fiber diet in DM care?	-0.009	0.702	-0.010	0.878	-0.001	0.337	-0.004	0.439	-0.010	0.846
Is foot care necessary in DM?	-0.008	0.642	0.008	0.178	0.034	**0.037**	-0.010	0.901	-0.009	0.769

Knowledge regarding complications of DM										
Unmanaged DM can cause eye problems or even blindness	0.008	0.178	-0.010	0.981	-0.010	0.889	-0.009	0.944	0.18	0.100
Uncontrolled DM can affect your kidneys	-0.006	0.534	-0.002	0.381	-0.010	0.839	0.013	0.777	-0.010	0.963
Uncontrolled DM can cause ischemic heart disease.	-0.009	0.715	-0.006	0.502	-0.010	0.974	-0.009	0.131	-0.003	0.414
Uncontrolled DM can cause stroke	-0.009	0.706	0.040	**0.026**	-0.009	0.722	0.018	0.703	-0.009	0.783

*R* ^2^: adjusted *R* squared; *p* < 0.05 is considered as significant.

## Data Availability

The data used to support the findings of this study are available from the corresponding author upon request.
